# One-month-old girl presenting with pseudohypoaldosteronism leading to the diagnosis of *CDK13*-related disorder: a case report and review of the literature

**DOI:** 10.1186/s13256-019-2319-x

**Published:** 2019-12-29

**Authors:** Renata Yakubov, Asaly Ayman, Adi Klein Kremer, Machiel van den Akker

**Affiliations:** 1 Department of Pediatrics, Nephrology Unit, Hillel Yaffe Medical Center, Ha-Shalom Street, 38100 Hadera, Israel; 2 Department of Pediatrics, Queen Paola Children’s Hospital, Antwerp, Belgium; 3 Department of Pediatric Hematology Oncology, UZ Brussel, Brussels, Belgium

**Keywords:** Pseudohypoaldosteronism, *CDK13*-related disorder, Hyperkalemia, Craniofacial dysmorphic features

## Abstract

**Background:**

It is not uncommon that an infant with a disease of unknown etiology is presented to a physician. Facial dysmorphic features lead to a different diagnosis. It is a challenge to link the presentation to the newfound diagnosis.

**Case presentation:**

A 37-day-old Yemenite Jewish girl was presented to our institution with a clinical picture of pseudohypoaldosteronism due to abnormal facial features and a psychomotor developmental delay. Further investigation led to the diagnosis of *CDK13*-related disorder. According to the literature, *CDK13* has a key role in the cell cycle, but no interference with the aldosterone signaling pathway or electrolyte balance was described. No mutations in the previously described gene *NR3C2* (cytogenetic location 4q31.23), encoding the mineralocorticoid receptor, were found. Although the clinical presentation corresponded to pseudohypoaldosteronism type 1, we could not genetically confirm this.

**Conclusions:**

Probably pseudohypoaldosteronism was a coincidental finding in this girl with a *CDK13* mutation, but because only limited information is known about *CDK13*-related disorders, further investigation could be more informative to clarify this presentation.

## Introduction

Water balance and potassium homeostasis are critically regulated by the aldosterone hormone. Aldosterone binds to the mineralocorticoid receptor, which is expressed in the distal tubules of the kidney, the salivary and sweat glands, the intestinal epithelium, and in the airway epithelium in the lung and heart. Aldosterone binding to the mineralocorticoid receptor results in increased expression of the epithelial sodium channel (ENaC) and the Na^+^-K^+^-ATPase in principal cells of the distal tubule in nephrons, and potassium accumulated in principal cells is secreted into the urine by the renal outer medullary potassium channel. Aldosterone also mediates proton secretion into the urine by induction of H^+^-K^+^-ATPase in intercalated cells, leading to a net sodium-water reabsorption and potassium and proton secretion [1–3]. Pseudohypoaldosteronism (PHA) is a heterogeneous disorder characterized by salt wasting resulting from target organ unresponsiveness to mineralocorticoids. Because the principal effect of aldosterone is to promote potassium and hydrogen secretion, the condition typically presents in the neonatal period with hyperkalemia, metabolic acidosis, and an elevated plasma aldosterone concentration because of loss of negative feedback. We present a case of an infant with the classic presentation of PHA but clinical features that raised suspicion of other problems.

## Case presentation

A 37-day-old Yemenite Jewish girl was seen in the emergency department of our institution for ongoing restlessness and feeding refusal. The girl was born at term by uncomplicated vaginal delivery after a normal pregnancy. Besides folic acid, no other medications had been used. Her birth weight was 2.435 kg (standard deviation [SD], − 1.8); her length was 50 cm (SD, 0.3); and her head circumference was 34 cm (SD, − 1.4). The result of her physical examination was reported as normal. She was first child of healthy, nonconsanguineous Yemenite Jewish parents. Her family history was unremarkable. During the first weeks of life, she failed to gain weight, and gastroesophageal reflux disease was suspected. Empirically, Enfamil AR (Mead Johnson & Co., Chicago, IL, USA) and a proton pump inhibitor (omeprazole 10 mg twice daily) were started with moderate response.

At the emergency department, the girl was presented in stable condition with vital signs appropriate for her age (pulse 128 beats/minute, blood pressure 90/50 mmHg, respiratory rate 30 breaths/minute, normal peripheral perfusion) and normal hydration status. Her weight was 3.340 kg (SD, − 2.0); her length was 52 cm (SD, − 1.4); and her head circumference was 36 cm (SD, − 1.5). Her physical examination revealed craniofacial dysmorphism (low-set ears, micrognathia, flat midface, broad nasal bridge, telecanthus, epicanthus, thin upper vermillion) and hypotonia, but the remainder of her examination was unremarkable. No skin or musculoskeletal (including skull and vertebral column) abnormalities were noted. The result of her eye examination was normal.

Laboratory tests (see Table 1) revealed hyperkalemic metabolic acidosis. Because the patient’s serum anion gap was normal and she had no gastrointestinal loss of bicarbonate, type 4 renal tubular acidosis was the primary working diagnosis. Her elevated ammonia can be explained by hyperkalemia causing reduction of ammonia generation in the proximal tubule leading to impaired ammonia excretion responsible for the metabolic acidosis [4]. Hyperkalemic metabolic acidosis gave rise to the suspicion of congenital adrenal hyperplasia (CAH). Further testing revealed elevated levels of renin and aldosterone but normal levels of 17-hydroxyprogesterone and 21-α-hydroxylase. The result of a synacthen test to exclude late-onset CAH was normal. Radiologic evaluation showed no abnormalities of the kidneys and the adrenal glands. When CAH was ruled out, hyperkalemic metabolic acidosis together with high levels of renin and aldosterone made the diagnosis of PHA type 1 very likely. Treatment with kayexalate was initiated with gradual normalization of the potassium levels, and it was continued five times per week until it was stopped when the girl reached the age of 2 years.

**Table 1:** Laboratory test results at presentation

Test	Result	Range normal values
Blood		
Alanine transaminase	37 U/L	10–50 U/L
Aspartate transaminase	45 U/L	20–60 U/L
Glucose	88 mg/dl	60–105 mg/dl
Creatinine	0.3 mg/dl	0.2–0.6 mg/dl
Blood urea nitrogen	12 mg/dl	8–28 mg/dl
Sodium	139 mmol/L	135–148 mmol/L
Potassium	7.13 mmol/L	3.5–5.8 mmol/L
Chloride	108 mmol/L	96–111 mmol/L
Calcium	9.7 mg/dl	8.0–10.7 mg/L
Phosphate	7.3 mg/L	4.8–8.1 mg/L
Magnesium	2,2 mg/dl	1.6–2.6 mg/dl
Lactate	91.8 mg/dl	4.5–19.8 mg/dl
Ammonia	185 μg/dl	29–70 μg/dl
Blood gas (capillary)		
pH	7.20	7.34–7.43
HCO_3_^−^	19 mEq/L	19–24 mEq/L
Base excess	− 6 mEq/L	− 7 to − 1 mEq/L
Serum anion gap	12 mEq/L	8–16 mEq/L
Urine		
Sodium (Na^+^)	85.4 mmol/L	
Chloride (Cl)	81.1 mmol/L	
Potassium	4.5 mmol/L	
Fractional excretion of sodium	3.3%	1–2%
Fractional excretion of potassium	4.6%	10–30%
Urine anion gap	+ 8.8	negative
Hormones (blood)		
17-α-Hydroxyprogesterone	1.1 nmol/L	< 2.5 nmol/L
21-α-Hydroxylase	Normal	
Cortisol	26.5 μg/dl	5–25 μg/dl
Dehydroepiandrosterone	1.89 nmol/L	35–430 nmol/L
Renin	5.0 ng/ml/hour	0.5–3.9 ng/ml/hour
Aldosterone	> 1717 pg/ml	25–190 pg/ml

Considering the dysmorphic physical signs along with metabolic disturbances, we suspected a congenital anomaly. Chromosome analysis revealed a normal 46XX karyotype. Echocardiography and magnetic resonance imaging (MRI) of the brain (including cervical spine) revealed no abnormalities. The patient was followed closely in the pediatric nephrology and neurology clinics.

Whole-genome sequencing was requested, which revealed a missense mutation (c.2525A>G [p.Asn842Ser]) in the cyclin-dependent kinase 13 gene (*CDK13*) located on 7p14.1. Genetic counseling was provided, and the parents underwent genetic testing, which revealed normal *CDK13* genes. Advice for prenatal diagnosis was not given, owing to the very low risk of recurrence.

Over time, the girl’s psychomotor development was clearly delayed. At the age of 8 years, she is still hypotonic. The result of further neurologic examination was normal. Her head circumference is 50 cm (SD, − 1.3); her length is 123 cm (SD, − 0.8); and her weight is 21 kg (SD, − 1.4). She has had no seizures. She has moderate mental retardation and behavior problems. Her current medications are risperidone, methylphenidate, and clonidine. She has a normal diet and eats independently, but she needs assistance with many general daily activities. She receives appropriate education. She is regularly seen by a neurologist and a psychiatrist in the outpatient clinic.

## Discussion

PHA is a rare, inherited salt-wasting disorder that was first described by Cheek and Perry in 1958 as a defective renal tubular response to mineralocorticoids in infancy [5]. This clinical syndrome is caused by end-organ resistance of aldosterone, resulting in hyperkalemic metabolic acidosis with high serum aldosterone levels. PHA can be primary (hereditary) or secondary (acquired) [6].

The primary form is subclassified into type 1 and type 2. PHA type 1 can be divided into a renal form (autosomal dominant inheritance) and a systemic form (autosomal recessive inheritance). The renal form is more frequent and caused by inactivating mutations in the *NR3C2* gene encoding the aldosterone receptor [7], resulting in an absence of binding of aldosterone to the receptor [8]. To date, more than 100 mutations in this gene have been reported [2, 9–13]. The salt loss is restricted to the kidneys and is biochemically accompanied by hyperkalemic metabolic acidosis with elevated plasma renin and aldosterone levels. Generally, the clinical expression of this condition can vary widely. It typically has a mild course followed by spontaneous remission over time [14, 15]. The systemic form is caused by mutations in the *SSCNN1A*, *SCNN1B*, and *SCNN1G* genes encoding for the ENaC α, β, and γ subunits, respectively, resulting in impairment in channel activity. Mutations in the α subunit are the most common and could involve the ENaC of kidneys (cortical collecting tubules), respiratory airways, colon, and salivary and sweat glands. PHA type 2 (Gordon syndrome or familial hyperkalemic hypertension) shows consistent features of hypertension, hyperkalemia, hyperchloremia, and mild metabolic acidosis. The reabsorption of sodium and the renal function are normal. The renin is low, whereas the aldosterone level can be normal or mildly elevated. In this autosomal dominant type, mutations affecting *WNK4* kinase have been reported and are important in the regulation of ion transport in the kidney, behaving like a gain of function in the thiazide-sensitive NaCl cotransporter.

The secondary form (also termed “type 3”) may be caused by medications or may be seen with acute pyelonephritis, urologic malformations, and obstructive uropathy [16]. This secondary PHA is always transient.

In the presence of hyponatremia, hyperkalemia, and nongap metabolic acidosis, possible signs of adrenal insufficiency, it is important to exclude CAH. In cases of atypical external genitalia, an urgent karyotype must be done to determine the gender. To differentiate from PHA type 1, investigations should include plasma renin activity, aldosterone, 17-hydroxyprogesterone, dehydroepiandrosterone, cortisol levels, renal ultrasound scan, and a urine steroid profile [6].

To date, *CDK13*-related disorder has been reported in 44 patients [17], and although there is a wide phenotypic heterogeneity [18], all patients are characterized with developmental delay (motor and speech domains) and variable craniofacial features (hypertelorism, flat midface, broad nasal bridge, small mouth, low-set ears); most show mild to moderate intellectual disability. Other common findings are feeding difficulties (gastroesophageal reflux, velopharyngeal dysfunction), structural brain abnormalities (hypoplastic or absent corpus callosum, periventricular leukomalacia or gliosis, Chiari malformations), and structural heart defects (most commonly atrial or ventricular septal defects) [17]. Bostwick *et al.* reported three patients with renal abnormalities (duplicated or dilated collecting systems, fused renal ectopia) [18]. To the best of our knowledge, no metabolic abnormalities or electrolyte disturbances have been reported to be related to *CDK13* gene mutations.

Cyclin-dependent kinases are a family of serine threonine kinases and are involved in several key cellular processes that include regulation of the cell cycle, transcription, RNA splicing, neurogenesis, and apoptosis [19]. *CDK13* is involved in pre-mRNA splicing and is a component of the perinucleolar compartment, which is primarily detected in solid tissue-derived cancer cells and rarely present in normal cells [20].

*CDK13*-related disorder is inherited in an autosomal dominant manner, and all cases are the result of a *de novo CDK13* pathogenic variant. No individuals with *CDK13*-related disorder are known to reproduce [21]. Although no clinical diagnostic criteria have been published, the clinical findings of developmental delay and facial dysmorphic features are suggestive, and the diagnosis of *CDK13*-related disorder primarily depends on molecular genetic testing. Of the 44 patients reported, 36 were found to have missense mutation affecting the protein kinase domain of *CDK13*. Of these 36, 20 affected amino acid residue 842, and 18 had the same mutation (p.Asn842Ser) as our patient [17]. Structural anomalies of the heart and brain are common in the patients with mutations affecting amino acid residue 842, although this was not the case in our patient [17]. Workup for suspected *CDK13* gene mutations should include diligent physical examination; MRI of the brain; echocardiography; and consultations with a neurologist, cardiologist, dietitian, and geneticist. More extensive research may be required (such as radiology of the spine). The follow-up is very individual-dependent but includes neurological follow-up, as well as support in daily life, such as suitable schooling and proper nutrition [22].

According to the literature, *CDK13* has a key role in the cell cycle, but no interference with the aldosterone signaling pathway or electrolyte balance has been described.

No mutations in the previously described *NR3C2* gene (cytogenetic location 4q31.23), encoding the mineralocorticoid receptor, were found in our patient. Although the clinical presentation corresponded to PHA type 1, we could not genetically confirm this.

## Conclusion

We report a case of a child presenting with PHA with a *CDK13* gene mutation. It is possible that the PHA at presentation was a coincidental finding in this girl with a *CDK13* mutation, but because only limited information is known about *CDK13*-related disorders, further investigation could be more informative to clarify this.

## Data Availability

The data are noted in the report. Additional information can be requested from the corresponding author.
